# Potential lipolytic regulators derived from natural products as effective approaches to treat obesity

**DOI:** 10.3389/fendo.2022.1000739

**Published:** 2022-09-13

**Authors:** Xi-Ding Yang, Xing-Cheng Ge, Si-Yi Jiang, Yong-Yu Yang

**Affiliations:** ^1^Department of Pharmacy, Second Xiangya Hospital of Central South University, Changsha, China; ^2^Phase I Clinical Trial Center, The Second Xiangya Hospital of Central South University, Changsha, China; ^3^Xiangxing College, Hunan University of Chinese Medicine, Changsha, China; ^4^Department of Pharmacy, Medical College, Yueyang Vocational Technical College, YueYang, China; ^5^Hunan Provincial Engineering Research Central of Translational Medical and Innovative Drug, The Second Xiangya Hospital of Central South University, Changsha, China

**Keywords:** adipose tissue, insulin resistance, lipolysis, natural product, obesity

## Abstract

Epidemic obesity is contributing to increases in the prevalence of obesity-related metabolic diseases and has, therefore, become an important public health problem. Adipose tissue is a vital energy storage organ that regulates whole-body energy metabolism. Triglyceride degradation in adipocytes is called lipolysis. It is closely tied to obesity and the metabolic disorders associated with it. Various natural products such as flavonoids, alkaloids, and terpenoids regulate lipolysis and can promote weight loss or improve obesity-related metabolic conditions. It is important to identify the specific secondary metabolites that are most effective at reducing weight and the health risks associated with obesity and lipolysis regulation. The aims of this review were to identify, categorize, and clarify the modes of action of a wide diversity of plant secondary metabolites that have demonstrated prophylactic and therapeutic efficacy against obesity by regulating lipolysis. The present review explores the regulatory mechanisms of lipolysis and summarizes the effects and modes of action of various natural products on this process. We propose that the discovery and development of natural product-based lipolysis regulators could diminish the risks associated with obesity and certain metabolic conditions.

## 1 Introduction

Obesity is excessive lipid accumulation in adipose tissue. It is caused by an imbalance between energy intake and energy consumption. According to the World Health Organization (WHO), more than 650 million adults over 18 years of age were obese as of 2016 (1). Obesity is a risk factor for cardiovascular disease (CVD), insulin resistance (IR), type 2 diabetes mellitus (T2DM), hypertension, dyslipidemia, and certain cancers (2). Increased adipocyte number (hyperplasia) and size (hypertrophy) are morphological manifestations of obesity (3). Adipose tissue is classified into three distinct types: white (WAT), brown (BAT), and beige (4). WAT stores excess energy in the form of triglycerides (TG), whereas BAT and beige adipose tissues catabolize TG into heat (5). Adipose tissue also functions as an endocrine organ and filler tissue and cushions, supports, and insulates the body (6).

WAT is generally considered a ‘troublesome and excessive tissue’. Body weight may be lost *via* intermittent fasting, medication, exercise, or surgery (7). However, it is uncertain whether these approaches maintain weight loss or have unacceptable side effects in the long term. Exercise appears to be an effective weight loss method, although its efficacy depends largely on its duration, frequency, and intensity (8). The administration of certain drugs is promising for obesity prevention and treatment. Intermittent fasting, drugs, and exercise decompose TG faster than they are synthesized in the adipocytes. Hence, pharmacological and nutritional enhancements of this process are potential strategies for weight loss and the prevention of obesity-related metabolic syndrome.

The reservoir effect of WAT protects other tissues against the toxic effects of glycolipids associated with excess energy storage. Adipocytes have limited lipid storage capacity and can hold no additional TG when their volume expands beyond a critical point. At this stage, the adipose tissue becomes dysfunctional. This condition is observed in patients with insulin resistance, T2DM, and obesity and is manifested by decreased TG synthesis and excess free fatty acid (FFA) release (9). In cases of adipose tissue dysfunction, certain compounds improve whole-body energy metabolism by inhibiting lipolysis.

Natural products are vital sources of lead compounds and are important in drug discovery. Several natural products are widely used in obesity treatment (10). Various natural products (11–14), including flavonoids, alkaloids, and terpenoids control obesity by stimulating lipolysis, inhibiting adipogenesis and lipogenesis, and promoting energy expenditure. However, the activities and mechanisms of natural products in modulating lipolytic activity have not yet been systematically summarized. In the present review, from a lipolysis perspective, we describe the biosynthesis and metabolism of TG in adipose tissue and review the regulatory mechanisms of lipolysis. Furthermore, we summarize a wide diversity of plant secondary metabolites that have demonstrated anti-obesity effects *via* the promotion of lipolysis. We also focus on the progress of research on inhibitors of lipolysis with different mechanisms of action in adipose tissue dysfunction. This review provides insight into the precise biochemical and molecular mechanisms by which plant secondary metabolites inhibit the onset and/or progression of obesity and, by extension, its related co-morbidities. In addition, it highlights the potential of lipolysis as a therapeutic target for obesity and its complications.

## 2 Triglyceride biosynthesis and metabolism

**Figure 1:**
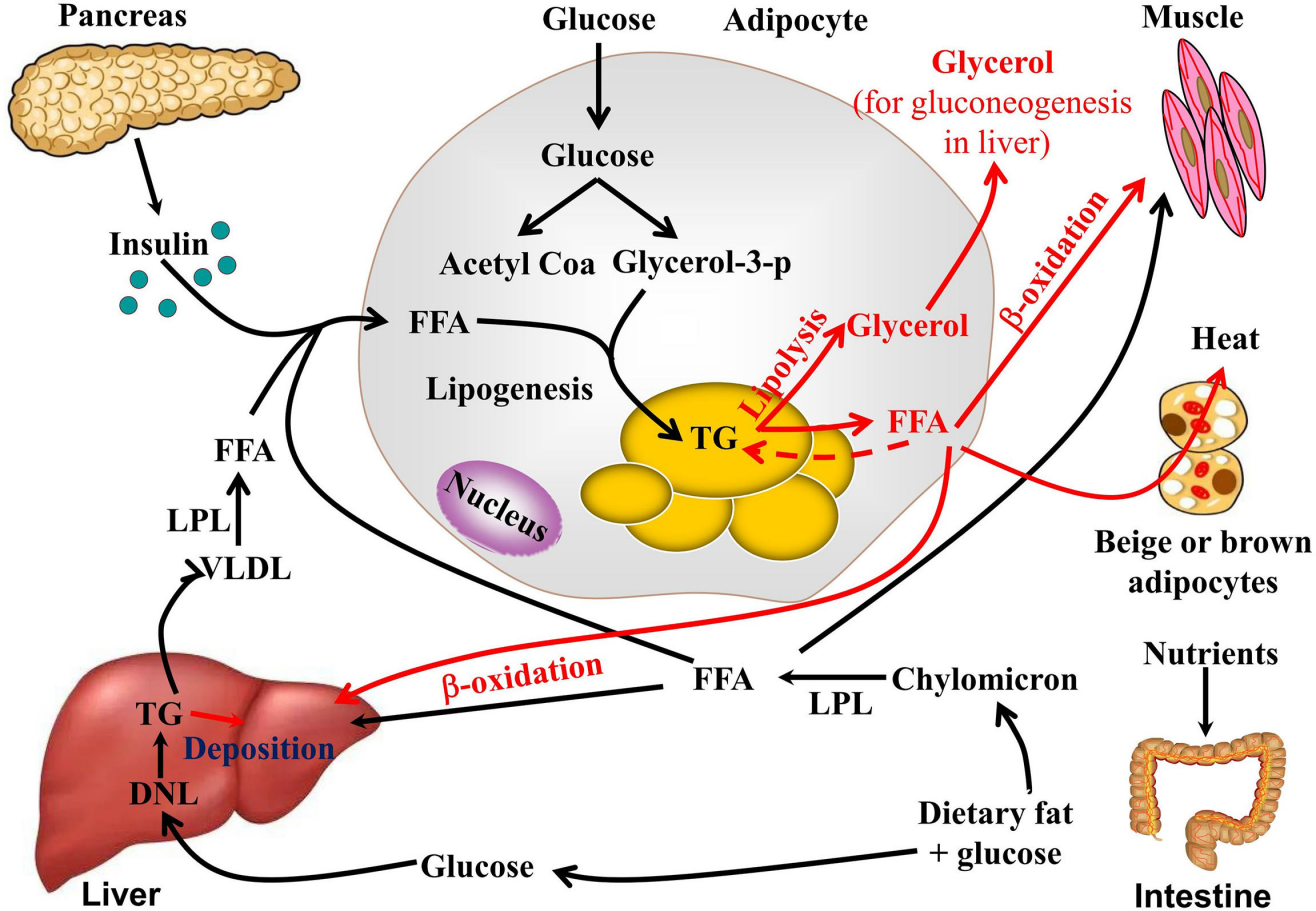
TG synthesis and metabolism.

Adipose tissue, the liver, and skeletal muscle are the mains organs responsible for the regulation of lipid metabolism. TG biosynthesis and decomposition (lipolysis) in WAT equilibrate lipid metabolism. After feeding, glucose and lipids from food are absorbed in the intestine in the form of chylomicrons and enter the bloodstream. The chylomicrons are then hydrolyzed into FFAs by lipoprotein lipase and absorbed and utilized by adipocytes and liver and muscle tissue. Insulin is secreted by β-cells in the pancreas and promotes FFA and glucose uptake, while insulin inhibits lipolysis *via* lipase inhibition. Adipocytes absorb excess FFA and glucose and produce TG as an energy storage form (15). During this process, adipogenesis and lipogenesis increase, while lipolysis, thermogenesis, and browning decrease. *De novo* lipogenesis involves TG biosynthesis and occurs in the adipocytes and liver. To maintain normal blood glucose levels, the liver converts excess glucose into glycogen and stores it in liver cells, or hepatocytes, which can also synthesize TG through the *de novo* TG synthesis pathway. TG subsequently is transported from the liver to adipose tissue by very low-density lipid (VLDL) (16). An important contributor to hepatic fat accumulation is the insufficient hepatic export of TG in the form of VLDL particles. TG synthesis and metabolism are illustrated in **Figure 1**.

During fasting and starvation, TG is decomposed into glycerol and FFA (17). Adipose triglyceride lipase (ATGL), hormone-sensitive lipase (HSL), and monoacylglycerol lipase (MAGL) hydrolyze TG to FFAs and glycerol. The glycerol is used to make glucose in gluconeogenesis. FFAs are then released into circulation, where they are utilized by the peripheral tissues and/or re-esterified into TG in the adipocytes. Skeletal muscle and the liver are the most important organs involved in FFA metabolism *via* β-oxidation and subsequent ATP generation. Mitochondrial-rich beige adipose tissue or BAT are the major sites responsible for non-shivering thermogenesis in mammals. Cold exposure, β-adrenergic receptor (β-AR) agonists, peroxisome proliferator-activated receptor-γ, and exercise can induce the browning of WAT. FFA produced by lipolysis is also absorbed and utilized by beige adipocytes or BAT through UCP-1-dependent shiver-independent thermogenesis.

## 3 Lipolysis and its mechanisms

Lipolysis is a finely regulated process mediated by the consecutive actions of ATGL, HSL, and MAGL. ATGL or HSL first hydrolyzes TG to diglycerides and FFA. HSL then hydrolyzes diglycerides to monoglycerides and FFA. MAGL then hydrolyzes monoglycerides to glycerol and FFA (18). Lipid droplet autophagy or lipophagy is a complementary cellular lipid breakdown pathway (19). Sex, age, physical activity, fat deposit location, and genetic variation regulate basal lipolytic activity in adipocytes (20). The proinflammatory cytokines TNF-α (21), IL-6 (22), and IL-1β (23) as well as lipopolysaccharide (LPS) (24) and hypoxia (25) may induce TG lipolysis. Lipid droplet-associated proteins (LDAPs) (26), cyclic guanosine monophosphate dependent-protein kinase G (cGMP-PKG) (8), mitogen-activated protein kinase (MAPK) (27), and adenosine 5’-monophosphate (AMP)-activated protein kinase (AMPK) (28) are also implicated in TG lipolysis.

### 3.1 LDAPs

Lipid droplets (LD) are dynamic lipid storage organelles surrounded by single layers of polar and amphipathic phospholipids and structural proteins. They are now considered major fat storage, lipid secretion, and lipolysis regulators (29). The perilipins, including perilipin1, perilipin2, and perilipin5, as well as the cell death-inducing DNA fragmentation factor alpha (DFFA)-like effector (CIDE) family proteins, including Cidea, Cideb, and Cidec/Fsp27, have emerged as key lipolysis regulators (30, 31). Perilipin1 is a scaffold for organized protein-protein interactions on LD surfaces. It binds CGI-58 and suppresses HSL translocation to LD under basal conditions. During stimulatory conditions, however, phosphorylation causes perilipin to dissociate from CGI-58. The free CGI-58 then binds phosphorylated ATGL and co-activates TG hydrolysis (32). Perilipin phosphorylation recruits HSL from the cytosols to the surfaces of the LDs, and diglycerides are then hydrolyzed (26). FSP27-deficient cells exhibite increased basal lipolysis and reduced lipid storage capacity (33). The mechanisms by which perilipin1 regulates lipolysis are generally understood. However, the roles and mechanism of perilipin2, perilipin5, and the CIDE family in lipolysis remain to be elucidated.

### 3.2 cAMP-PKA pathway

*In vivo*, dynamic lipolysis processes are mainly regulated by hormones, such as catecholamines, ghrelin, adiponectin, and insulin. Under conditions of fasting, cold stress, and other compound treatment, norepinephrine is released from sympathetic nerve terminals. β-AR agonists, such as epinephrine, norepinephrine, and dopamine, upregulate cyclic AMP (cAMP) by linking various AR subtypes to the G-protein receptor complex that controls adenylate cyclase in the cell membrane. Thereafter, protein kinase A (PKA) is activated by cAMP (34). PKA phosphorylates both HSLs at Ser563, Ser659, and Ser660, thereby activating them and promoting their translocation from the cytoplasm to the surfaces of LDs (35).

cAMP degradation is mediated by phosphodiesterase (PDE). Insulin inhibits lipolysis mainly by activating the phosphoinositide 3-kinase/protein kinase B/PDE 3B (PDE3B) pathway, which leads to p-HSL and p-perilipin dephosphorylation (36). In addition, ligands of Gi protein-coupled receptors, such as succinic acid, nicotinic acid, beta-hydroxybutyric acid, and neuropeptide Y, inhibit the formation of cAMP by binding to their receptors, thereby exerting an anti-lipolytic effect.

### 3.3 cGMP-PKG

Cyclic guanosine 3’5’-monophosphate (cGMP) is an important intracellular secondary messenger of hormone-induced lipolysis. Atrial and b-type natriuretic peptides are nitric oxide (NO) donors that stimulate lipolysis in adipocytes *via* the cGMP/PKG pathway (8). PKG phosphorylates proteins associated with lipolysis, including HSL and perilipin, thereby promoting TG breakdown (37). The cGMP is also involved in TNF-α (iNOS/NO/GC/cGMP-dependent pathway)- and endothelin-1 (GC/cGMP/Ca^2+^/ERK/CaMKIII signaling pathway)-induced lipolysis in adipocytes (38–40). Few studies have reported on the involvement of cGMP/PKG in lipolysis regulation. Moreover, the roles of cGMP/PKG in lipolytic enzymes regulation and LDAPs remain to be clarified.

### 3.4 Mitogen-activated protein kinase

The mitogen-activated protein kinase (MAPK) family, which including extracellular signal-regulated kinases (ERKs), jun aminoterminal kinase (JNK), and p38 mitogen-activated protein kinases (p38) plays vital roles in adipogenesis and lipolysis. (β−AR) stimulation by catecholamine activates ERK1/2, which is sufficient to induce lipolysis by direct HSL phosphorylation at Ser600. JNK regulates lipolysis. JNK1/2 deficiency accelerates basal lipolysis in mouse adipocytes (41). The MEK1/2-ERK1/2 pathway controls TNF-α-stimulated lipolysis in human adipocytes (42).

### 3.5 AMPK pathway

AMPK is a Ser/Thr protein and an important regulatory sensor of cellular energy metabolism. Activated AMPK inhibits sterol regulatory element binding protein-1, CCAAT/enhancer binding protein alpha, peroxisome proliferator activated receptor gamma, and acetyl-CoA carboxylase (ACC). Hence, AMPK suppresses adipocyte differentiation (43). AMPK also phosphorylates ATGL Ser406, which promotes TG decomposition (44). However, the roles of AMPK in regulating TG lipolysis in adipocytes are controversial. AMPK may phosphorylate HSL at Ser565 to inhibit phosphorylation at HSL Ser660 and Ser563. In this manner, it reduces HSL activity and suppresses lipolysis (45). AMPK is implicated in chaperone-mediated autophagy which selectively degrades perilipins and initiates lipolysis (46). Therefore, proteins and signaling pathways that modulate AMPK expression and activity, such as SIRT (47) and SIRT3 (48), mobilize TG in adipocytes.

**Figure 2:**
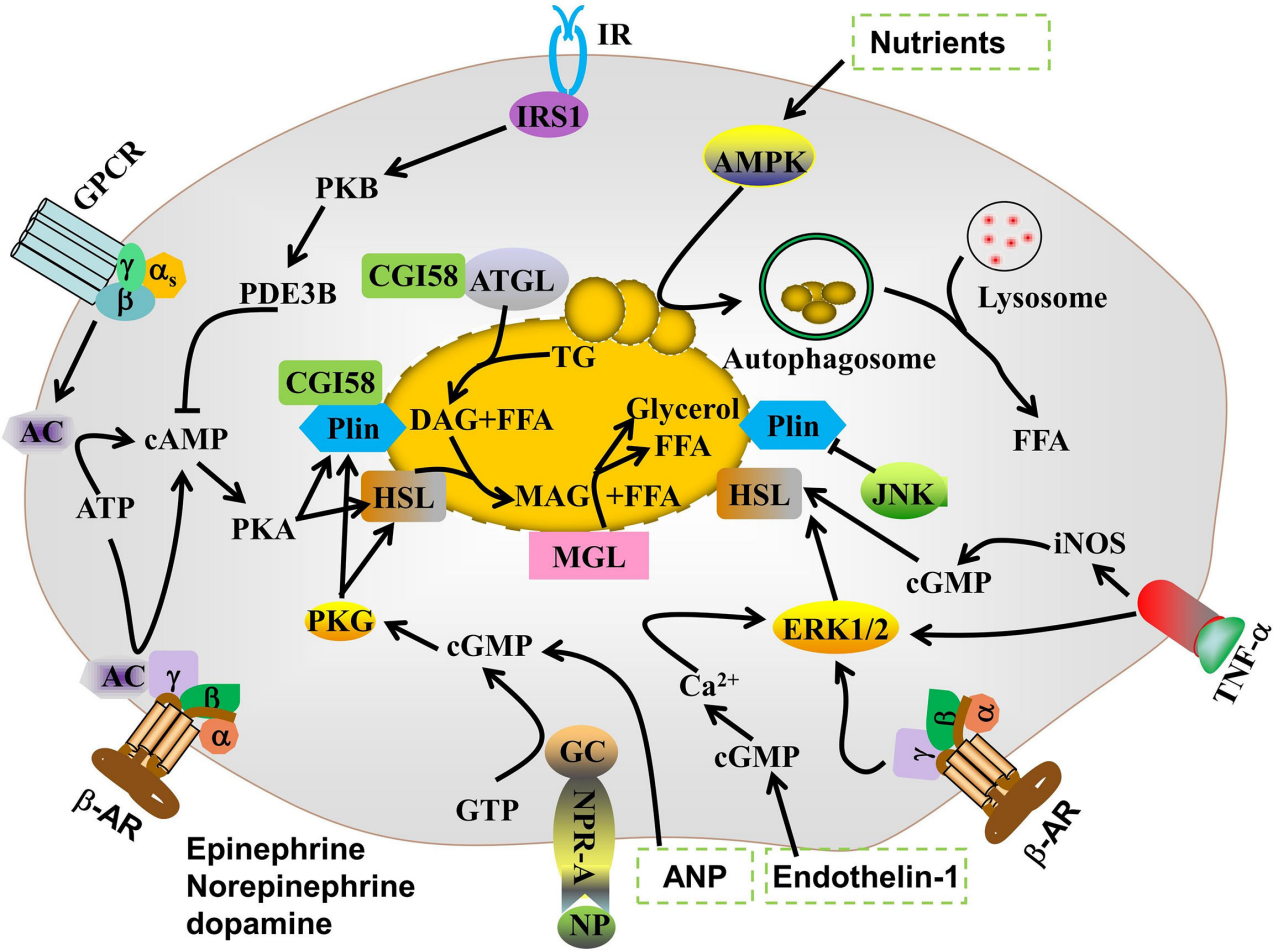
Signaling pathways regulating lipolysis in adipocytes.

Protein kinase C (49), Ca^2+^ (50), inositol hexakisphosphate kinase-1 (51), transient receptor potential vanilloid channels (38, 52), and endoplasmic reticulum (ER) stress (53) regulate lipolysis in adipocytes either alone or by interacting with the aforementioned signaling pathways (**Figure 2**).

## 4 Natural products involved in lipolysis

The structural diversity of natural products determines their wide range of pharmacological activity. Natural products may be used to treat obesity and its associated metabolic diseases. Traditional and complementary medicines including various herbs and extracts have been widely used to prevent and treat metabolic disorders (54, 55). Flavonoids (56), alkaloids (57), terpenoids (58), and polyphenols (59) stimulate lipolysis in adipocytes, thereby causing weight loss and improving metabolic status. Their modes of action involve the PKA-HSL, PKC, AMPK, MAPK, and other signaling pathways.

### 4.1 Natural products promote lipolysis

#### 4.1.1 Flavonoids

Flavonoids comprise a large family of natural substances sharing a molecular structure characterized by at least one phenolic ring. Flavonoids are reputed for their health benefits. Epigallocatechin-3-gallate (EGCG) is a polyphenolic catechin in green tea that improves the lipid prolife and reduces body weight (60). EGCG inhibits adipogenesis and adipocyte differentiation, reduces energy intake, and increases energy expenditure and lipolysis (61, 62). EGCG-stimulated lipolysis is mediated by activating HSL (63), ERK1/2 (64), and p-AMPK (65). Lipophagy is also associated with EGCG-induced lipolysis. Rab7 knockdown attenuates EGCG-dependent lipid reduction (65). However, a clinical trial demonstrated no effect of EGCG on obesity reduction, lipolysis, or white adipocyte browning in humans (66).

Kaempferol (67), apigenin (68), genistein (69), morusin (70), medicarpin (71), and myricetin (72) commonly occur in fruits, vegetables, and tea. These flavonoids have anti-obesity and pro-lipolysis efficacy. Elevated lipolysis upregulates thermogenic genes and increases mitochondrial biogenesis by supplying FFAs for mitochondrial β-oxidation. Apigenin activates lipolysis *via* the ATGL/FOXO1/SIRT1 pathway and increases FFA consumption by upregulating fatty acid oxidation (FAO) (AMPK/ACC), thermogenesis, and browning (UCP-1, PGC-1α) (68). Lipolysis is also associated with activated BAT or beiging which is regarded as an alternative strategy against diet-induced obesity. Xanthohumol (73), apigenin (68), and EGCG (65) inhibit adipogenesis, stimulate adipocyte lipolysis, and may act as browning or beiging agents because they upregulate the thermogenic protein UCP1 (**Table 1**).

**Table 1:** Lipolytic effects and modes of action of flavonoids.

Compound	Model	Concentration	Effect	Mechanism	Reference
EGCG	3T3-L1 adipocytes; C3H10T1/2 cells	10 µM	Adipogenesis inhibition Lipophagy activation and adipocyte browning	Increasing p-AMPK Lipophagy mediates EGCG-induced lipolysis	Kim et al. (65)
	3T3-L1 adipocytes	10 µM	Lipolysis promotion	Increasing HSL	Lee et al. (63)
	Rat primary adipocytes	2.79 µM	Lipolysis promotion	Increasing p-ERK1/2	Ogasawara et al. (64)
Kaempferol	3T3-L1 adipocytes	60 μM	Lipolysis promotion Adipogenesis inhibition	Increasing ATGL and HSL	Torres-Villarreal et al. (67)
Apigenin	HFD-Fed mice	0.04%	Increasing lipolysis, thermogenesis, and browning	Increasing ATGL, SIRT1, and p-AMPK	Sun et al. (68)
Myricetin	3T3-L1 adipocytes	50 and 100 μM	Increasing lipolysis	Decreasing perilipin1 Increasing p-p38 and p-JNK	Wang et al. (72)
Genistein	Primary rat adipocytes	0.1 and 1 mM	Increasing lipolysis	PKA-mediates, genistein-induced lipolysis	Szkudelska et al. (69)
Morusin	3T3-L1 and primary adipocytes	5, 10 and 20 μM	Lipolysis promotion Adipogenesis inhibition	Increasing HSL, ATGL, and p-perilipin expression	Lee et al. (70)
Medicarpin	BAT cells	(10 μM)	Lipolysis promotion	PKA-mediates, medicarpin-induced lipolysis	Imran et al. (71)
Xanthohumol	3T3-L1 and primary human adipocytes.	25 μM	Adipogenesis suppression Increasing lipolysis and white adipocyte beiging	AMPK signaling pathway mediates lipolysis	Samuels et al. (73)

#### 4.1.2 Alkaloids

Consumption of coffee, ephedrine, or capsaicin increases lipolytic responses, raise metabolic rates, and increase energy expenditure and weight loss (74, 75). Caffeine is the main alkaloid in tea, coffee, and cacao. It decreases body fat, improves glucose tolerance and insulin sensitivity (76), and increases lipolysis by raising cAMP levels and upregulating lipolytic enzymes (77). Ephedrine is an α- and β-adrenergic receptor agonist with efficacy as a bronchodilator. It also activates the β-adrenergic receptors, contributing to lipolysis (78). Capsaicin analogs significantly increase cAMP levels and PKA activity in BAT (79). While ephedrine, caffeine, capsaicin, and synephrine strongly induce lipolysis, they are also associated with various cardiovascular and gastrointestinal side effects when they are administered for weight loss (80). Therefore, novel lipolytic compounds with minimal adverse reactions merit further investigation.

Berberine (BBR) is an isoquinoline alkaloid derived from the Chinese herb *Coptis chinensis*. It has anti-obesity, anti-diabetic, and anti-hyperlipidemic efficacy. BBR stimulates basal lipolysis in 3T3−L1 adipocytes by upregulating ATGL *via* the AMPK pathway (81, 82). However, Zhou et al. found that BBR attenuates isoprenaline-stimulated lipolysis in 3T3−L1 adipocytes by reducing phosphodiesterase-3B and -4 inhibition, thereby decreasing cAMP production and inhibiting HSL activation (83). Trigonelline (*N*-methylnicotinic acid) is a pyridine derivative that increases brown and beige fat-specific markers as well as mitochondrial biogenesis in 3T3-L1 adipocytes (57). Trigonelline as well as cordycepin from *Cordyceps militaris* promotes white adipocytes beiging and browning and increases lipolysis by various mechanisms (57, 84) (**Table 2**).

**Table 2:** Lipolytic effects and modes of action of alkaloids.

Compound	Animal or cell model	Concentration	Effect	Mechanism	Reference
BBR	Differentiated porcine adipocytes	10-40 μM	Lipolysis and FFA oxidation promotion	Increasing p-ATGL Decreasing perilipin AMPK mediates BBR-induced lipolysis	Yang et al. (82)
Trigonelline	3T3-L1 cells	75 μM	Promoting lipolysis, browning, and FFA oxidation Decreasing adipogenesis and lipogenesis	β3-AR/PKA activation PDE4 inactivation	Choi et al. (57)
Capsaicin	3T3-L1 cells	10 μM	Promoting lipolysis	Increasing HSL and UCP2	Lee, et al. (85)
	HFD-Fed transient receptor potential vanilloid 1 deficient (TRPV1^-/-^) mice 3T3-L1 cells	Animal: chow plus 0.01% capsaicin Cell: 1 μmol/L	Promoting lipolysis	TRPV1 mediates capsaicin-induced lipolysis	Chen, et al. (86)
Caffeine	SD rats	5 mg/kg	Promoting lipolysis	N.A.	Kobayashi-Hattori et al. (87)
Cordycepin	Animal: S-D rats Cell: 3T3-L1 cells	Animal: 12.5, 25, and 50 mg/kg Cell: 1.563-25 μg/mL	White adipocyte beiging and browning Blocking lipid droplet formation and promoting lipid droplet degradation	Decreasing Fsp27, perilipin 3, perilipin 2, Rab5, Rab11, CGI-58 and perilipin 1 Increasing ATGL	Xu et al. (84)

HFD, high-fat diet; N.A., not available.

#### 4.1.3 Terpenoids

Terpenoids comprise five-carbon isoprene units and have diverse effects on obesity and its associated metabolic diseases. Triterpenoids include 18β-glycyrrhetinic acid (18β-GA) (88), ursolic acid (89), acetyl-keto-β-boswellic acid (AKBA) (90), alisol A 24-acetate (AA-24-a) (91), celastrol (92), and betulinic acid (93). All of these reduce neutral lipids in the cytosol and increase FFA release. Madecassoside (94), tanshinone 1 (95), triptolide (58), crocin (96), guggulsterone (97), bilobalide (98), α-cubebenoate (99, 100), betulinic acid (93), fucoxanthinol (101), widdrol (102), ginkgolide C (103), and illudins C2 and C3 (104) could all potentially treat obesity either by inhibiting adipocyte differentiation and lipogenesis or by increasing lipolysis. The LDAP (88–90), PKA (89, 90), AMPK (96, 98), and PKC-MEK-ERK (102) pathways are involved in the lipolytic mechanisms induced by these compounds (**Table 3**).

**Table 3:** Lipolytic effects and modes of action of terpenoids.

Compound	Animal or cell model	Concentration	Effect	Mechanism	Reference
18β-GA	3T3-L1 cells	40 μM	Inhibiting adipogenic differentiation Increasing lipolysis	Increasing HSL, ATGL, perilipin and p-HSL expression	Moon et al. (88)
Ursolic acid	Primary rat adipocytes	25 and 50 μM	Increasing lipolysis	Increasing HSL translocation and ATGL expression Decreasing perilipin1 PKA participates in lipolytic action of UA	Li et al. (89).
AKBA	3T3-L1 adipocytes	30 μM	Increasing lipolysis	Increasing ATGL and HSL Decreasing perilipin	Liu et al. (90)
Betulinic acid	Rat adipocytes	10 and 25 μM	Increasing lipolysis	Decreasing PDE activity	Kim et al. (93)
AA-24-a	3 T3-L1 cells	30, 40 and 50 μM	Increasing lipolysis	PKA- and ERK- mediated AA-24-A-promote lipolysis	Lou et al. (91)
Celastrol	C57BL/6N mice fed HFD	7.5 mg/kg/d for 21 d	Inhibiting lipogenesis Increasing lipolysis and thermogenesis	Inhibiting endoplasmic reticulum (ER) stress	Luo et al. (92)
	3T3-L1 adipocytes	400 nM	Inhibiting adipocyte differentiation and adipogenesis	N.A.	Choi et al. (105)
Tanshinone 1	Immortalized brown adipocytes (iBAs) and differentiated C3H10T1/2 cells	15 μM	Reducing HFD-induced obesity Activating brown adipocytes Increasing lipolysis and browning	Increasing HSL and p-AMPK	Jung et al. (95)
*Cis*-Guggul-sterone	3T3-L1 adipocytes	25 and 50 μM	Inhibiting lipid content Increasing lipolysis	Increasing p-ERK1/2	Yang et al. (97)
Madecas- soside	KKay/TaJcl obese diabetic mice	40 mg/kg/d	Inhibiting lipogenesis. Promoting lipolysis and thermogenesis	Increasing p-HSL, p-AMPK	Sun et al. (94)
Triptolide	Cell: 3T3-L1 and porcine adipocytes Animal: C57BL/6J fed HFD	Cell: 10 nM Animal: 0.2 mg/kg for 7 wks	Reducing fat tissue accumulation Increasing heat production Increasing lipolysis	P53-mediated ATGL transcription responsible for triptolide-induced lipolysis	Wang et al. (58)
Crocin	Cell: 3T3-L1 adipocytes Animal: db/db mice	Cell: 20 μM Animal: 20 mg/kg/d	Increasing lipolysis Inhibiting preadipocyte differentiation and adipogenesis	AMPK mediates crocin-trigged lipolysis	Gu et al. (96)
Bilobalide	3T3-L1 adipocytes	25 and 100 μM	Inhibiting preadipocyte differentiation and adipogenesis Increasing lipolysis	Increasing ATGL, pHSL, pACC1/ACC1, and pAMPK/AMPK	Bu et al. (98)
α-Cubebe- noate	Primary adipocytes and 3T3-L1 adipocytes	10, 20, and 30 μg/mL	Inhibiting adipogenesis and lipogenesis Increasing lipolysis	Increasing pHSL, ATGL, and p-perilipin	Bae et al. (99)
α-Cubebenol	3T3-L1 adipocytes	7.5, 15, and 30 μg/mL	Inhibiting adipogenesis Increasing lipolysis	Increasing cAMP, ATGL, p-perilipin, and p-HSL Decreasing perilipins and PDE4	Lee et al. (100)
Illudins C2 and C3	3T3-L1 adipocytes	5 and 10 μM	Suppressing adipogenesis Increasing lipolysis	PKA and ERK mediate illudins C2 and C3-stimulated lipolysis	Kim et al. (104)
Fuco- xanthinol	3T3-L1 adipocytes	5 and 10 μM	Decreasing TG content Increasing lipolysis	Increasing ATGL, pHSL, pACC1/ACC1, and pAMPK/AMPK Decreasing CGI-58, ATGL, p-HSL, and perilipin	Yoshikawa et al. (101)
Widdrol	3T3-L1 adipocytes	10-25 μg/mL	Increasing lipolysis	PKC and MEK/ERK pathway mediated Widdrol-induced lipolysis	Jeong et al. (102)
Ginkgolide C	3T3-L1 adipocytes	10, 30 and 100 μM	Suppressing adipogenesis and promoting lipolysis	Increasing ATGL, p-HSL, and p-AMPK	Liou et al. (103)

N.A., not available.

Celastrol and triptolide are the main bioactive constituents in the root of *Tripterygium wilfordii*. The administration of celastrol and triptolide reduces body and fat weight, suppresses lipogenesis (58, 92), increases heat production in BAT, and enhances lipolysis (58). Celastrol rapidly lowers body weight by covalently inhibiting GRP78 chaperone activity and disconnecting ER stress signal transduction (92). Elevated lipolysis induced by triptolide is mediated by p53 which directly binds and promotes the transcription of the ATGL promoter (58). Although triptolide and celastrol have good anti-obesity efficacy, their potential toxicity must be established.

#### 4.1.4 Other compounds

Resveratrol (RSV) (106), 2,4,5-trimethoxybenzaldehyde (2,4,5-TMBA) (11), raspberry ketone (RK) (107), cinnamaldehyde (108), lipoic acid (109), syringic acid (110), 6’-O-acetyl mangiferin (111), ferulic acid (112), and magnolol (113) have all demonstrated potential prophylactic and therapeutic efficacy against obesity. RSV directly affectes isoprenaline-stimulated lipolysis *in vitro* in fac cells from overweight humans (114). It also increases FFA and glycerol content in high-fat diet (HFD)-fed mice or 3T3-L1 adipocytes (106). Arrate et al. showed ATGL-mediated, RSV-induced lipolysis *in vivo* (115). However, a randomized, double-blind, crossover study revealed that RSV improved adipose tissue lipolysis and decreased plasma FFA and glycerol levels (116). This apparent contradiction in the anti-obesity effects of RSV in rodents and humans necessitates the re-evaluation of RSV as a putative anti-obesity drug.

RK has a structure resembling those of capsaicin and synephrine and can prevent HFD-induced obesity (117). 3T3-L1 adipocytes treated with 10 µM RK presented with elevated FAO and inhibition of lipid accumulation (118). Magnolol is the main bioactive compound in *Magnolia officinalis*. Its lipolytic effect is mediated by the calcium/calmodulin-dependent protein kinase (CaMK)/ERK1/2 signaling pathway and not by PKA (119). Magnolol may cause browning in white adipocytes and augment thermogenesis (113) (**Table 4**). Further research in the form of animal models is required to validate the lipolytic potential and clinical value of the foregoing compounds.

**Table 4:** Lipolytic effects and modes of action of other compounds.

Compound	Animal or cell model	Concentration	Effect	Mechanism	Reference
2,4,5-TMBA	3T3-L1 adipocytes	100 μg/mL	Suppressing differentiation and adipogenesis Increasing lipolysis	Reducing perilipin Increasing HSL	Wu et al. (11)
Raspberry ketone	Animal: ICR mice +HFD Cell: Primary adipocytes	Animal: 1) HFD including 0.5, 1, or 2% RK 2) HFD containing 1% RK Cell: 10^−3^ μM and 10^-4^ μM	Preventing obesity Increasing norepinephrine-induced lipolysis	Increasing HSL protein translocation	Morimoto (117).
	3T3-L1 adipocytes	10 μM	Increasing FAO and lipolysis Suppressing lipid accumulation	N.A.	Park et al. (118)
	3T3-L1 adipocytes	10, 20, and 50 μM	Inhibiting adipogenic and lipogenesis Increasing lipolysis	Increasing ATGL and HSL	Park et al. (120)
RSV	Human adipocytes	100 μM	Increasing isoprenaline-induced lipolysis Impairing insulin-mediated anti-lipolysis	N.A.	Gomez-Zorita et al. (114)
	Animal: C57BL/6J mice +HFD Cell: 3T3-L1 adipocytes	Animal: 15 mg/kg Cell: 20 μM	Promoting lipolysis Improving metabolic abnormalities	N.A.	Gong et al. (106)
	Cell: 3T3-L1 adipocytes, human SGBS adipocytes Tissue: fat pads from wild-type, ATGL^-/-^ and HSL^-/-^ mice	100 μM	Increasing basal-, isoproterenol-, and isoproterenol-stimulated lipolysis	ATGL mediates RVS-induced lipolysis	Lasa et al. (115)
Lipoic acid	3T3-L1 adipocytes	250 μM	Increasing lipolysis	cAMP-PKA mediates LA-induced lipolysis	Fernández-Galilea et al. (109)
Cinnamal- dehyde	Animal: Swiss albino mice fed HFD. Cell: 3T3-L1 adipocytes	Animal: 10 mg/kg/d for 14 wks Cells: 20 μM and 40 μM	Inhibiting preadipocyte differentiation and lipid accumulation in adipocytes Increasing lipolysis and browning	Increasing HSL Decreasing Plin1	Khare et al. (108)
Magnolol	Sterol ester (SE)-loaded 3T3-L1 preadipocytes	5-60 μM	Promoting lipolysis	CaMK/ERK mediate magnolol-induced lipolysis	Huang et al. (119)
	3T3-L1 adipocytes		Promoting lipolysis, browning, and thermogenesis	Increasing p-HSL, PKA, p-AMPK, Plin1	Parray, et al. (113)
Syringic acid	3T3-L1 adipocytes	1000 μmol/mL	Promoting lipolysis	N.A.	John et al. (110)
6’-O-acetyl mangiferin	3T3-L1 adipocytes	12.5, 25, and 50 μM	Promoting lipolysis	Increasing p-HSL, ATGL, and p-AMPK	Sim et al. (111)
Ferulic acid	3T3-L1 adipocytes	10 μM	Inhibiting lipogenesis and promoting lipolysis	Increasing p-perilipin, p-HSL	Kuppusamy et al. (112)

N.A., not available.

The lipolytic effects of the compounds above have already been established in *in vivo* or *in vitro* experiments. For compounds with pro-lipolytic activity tested only *in vivo*, preclinical pharmacodynamics and safety evaluations are required. In pharmacodynamics experiments, primary outcome measures, such as change in body weight, food intake, resting metabolic rate, blood lipids, and biochemistry, need to be tested. In addition to general and specific toxicities of drugs, the safety evaluation should pay special attention to liver and kidney toxicity caused by long-term use of lipolysis agonists, as well as pancreatic damage, insulin resistance, and cardiovascular events that may be caused by elevated FFA.

### 4.2 Natural products that inhibit lipolysis

Adipose tissue dysfunction increases circulating FFA levels. Elevated FFAs are often observed in patients with IR and T2DM (9). Impaired lipogenic capacity driven by insulin signaling and re-esterification of FFA with adipocytes results in impaired buffering capacity for FFA and high concentrations of circulating FFA (26). Long-term over-activation of lipolysis may promote lipid ‘overflow’ into the muscle, liver, endothelium, heart, and β-cells, thereby causing muscular/hepatic IR, CVD, and impaired insulin secretion (121). For example, adipocyte-derived FFA is involved in regulating hepatic energy metabolism (122). FFA impairs the insulin signaling pathway by forming diacylglycerol and ceramides and increases gluconeogenesis *via* the hepatic acetyl-CoA pathway in liver during diseased states (26, 123), which leads to TG accumulation in the liver. In patients with adipose tissue dysfunction, then, the inhibition of lipolysis may ameliorate IR- and obesity-associated metabolic diseases. Thiazolidinedione antidiabetic drugs improve insulin sensitivity and reduce circulating FFA levels by attenuating lipolysis and FFA release (124).

Curcumin (125), astragaloside IV (AS-IV) (126), and ilexgenin A (127) attenuate lipolysis by modulating the cAMP/PKA/HSL pathway. The inhibition of lipolysis in adipose tissue may improve hepatic insulin sensitivity (125, 126). Ginsenoside Rg5 (Rg5) suppresses lipolysis and inhibited IR in muscle (128). The foregoing findings suggest that a decrease in adipose tissue lipolysis mediated by natural bioactive components is a potentially efficacious therapy for hepatic IR and related disorders.

TNF-α is a proinflammatory cytokine expressed in adipose tissue that might link obesity and IR (129) and increases plasma FFA levels in obesity and T2DM (130). AS-IV (131), curcumin (132), emodin (133), eicosapentaenoic acid (EPA) (134), and phillyrin (135) attenuates TNF-α-induced lipolysis by suppressing p-ERK1/2 and reversing perilipin or p-perilipin downregulation. Rosmarinic acid (RA) (136, 137), RSV (116), BBR (83), cyanidin-3-O-β-glucoside (C3G) (138), dihydrodehydrodiisoeugenol (DDE) (139), carnosic acid (137), and piceatannol (140) may also inhibit lipolysis. The effects and mechanisms of these compounds are summarized in **Table 5**.

**Table 5:** Anti-lipolytic effects and mechanisms of various compounds.

Compound	Animal or cell model	Concentration	Effect	Mechanism	Reference
RA	3T3-L1 adipocytes	50 μM	Inhibiting adipogenesis and lipolysis	Decreasing p-HSL-ser660 and p-perilipin A	Rui et al. (136)
AS-IV	Animal: ICR mice fed HFD	Animal: 50 and 100 mg/kg	Inhibiting lipolysis and hepatic lipid deposition Improving glucose tolerance	Decreasing cAMP Increasing PDE3B, AMP, and Akt	Du et al. (126)
	3T3-L1 adipocytes	50, 100, and 200 μM	Inhibiting TNF-α-induced lipolysis and improving IR	Increasing perilipin Decreasing p-ERK1/2	Jiang et al. (131)
Curcumin	Adipose tissue Cells: 3T3-L1 adipocytes	0.1, 1, and 10 μM	Inhibiting lipolysis Reducing lipid deposition and IR in liver	Decreasing cAMP, p-HSL and ER stress Increasing AMP and PDE3B	Wang et al. (125)
	3T3-L1 adipocytes	20 μM	Inhibiting TNF-α or catecholamine-induced lipolysis	Decreasing p-ERK1/2, p-perilipin, and HSL translocation	Xie et al. (132)
Ilexgenin A	Adipose tissue	20 or 50 mg/kg	Inhibiting lipolysis and hepatic IR	Decreasing cAMP, pSer-660-HSL and ER stress Increasing AMP, PDE3B, pSer-565-HSL, and p-AMPK	Li et al. (127)
BBR	3T3-L1 adipocytes	10 μM	BBR-decreased isoprenaline- and noradrenaline-induced lipolysis	Reducing PDE inhibition	Zhou et al. (83)
RSV	Obese human	150 mg/d for 30 d	Inhibiting lipolysis Increasing muscle and decreasing hepatic lipid content	N.A.	Timmers et al. (116)
Piceatannol	3T3-L1 adipocytes, brown adipocyte, and WAT	25 and 50 μM	Inhibiting basal and isoprenaline-stimulated lipolysis	Autophagy mediated ATGL, CGI-58, and perilipin1 downregulation induced by piceatannol	Kwon et al. (140)
EPA	Primary rat adipocytes, 3T3-L1 adipocytes, and rat adipose tissue	100 and 200 μM	Inhibiting IL-6- and TNF-α-induced lipolysis	Increasing pSer565 HSL Decreasing ATGL	Lorente-Cebrián et al. (134)
C3G	3T3-L1 adipocytes	50 μM	Inhibiting high glucose-induced lipolysis	Increasing AMPK activity Decreasing FoxO1-mediated ATGL transcription	Guo et al. (138)
Emodin	3T3-L1 adipocytes	50 μM	Increasing glucose metabolism Decreasing TNF-α-induced lipolysis	Decreasing p-perilipin and p-ERK1/2	Zhang et al. (133)
DDE	3T3-L1 adipocytes or human subcutaneous adipocytes	1 and 10 μM	Inhibiting basal- and TNF-α-induced lipolysis	N.A.	Nehrenheim et al. (139)
Phillyrin	3T3-L1 adipocytes	20, 40, 80 μM	Increase in glucose uptake and decrease in TNF-α-induced lipolysis	Decreasing p-ERK1/2 Increasing perilipin	Kong et al. (135)
Rg5	Animal: ICR mice fed HFD Cells: 3T3-L1 adipocytes	Animal: 50 mg/kg Cells: 0.1, 1, 10 μM	Inhibiting lipolysis in adipocytes and IR in muscle	Decreasing cAMP and p-PKA Increasing PDE3B and AMP	Xiao et al. (128)
Carnosic acid	Human multipotent, adipose-derived stem cells	10 μM	Inhibiting isoprenaline-induced lipolysis	N.A.	Colson et al. (137)

N.A., not available.

## 5 Conclusions and perspectives

**Figure 3:**
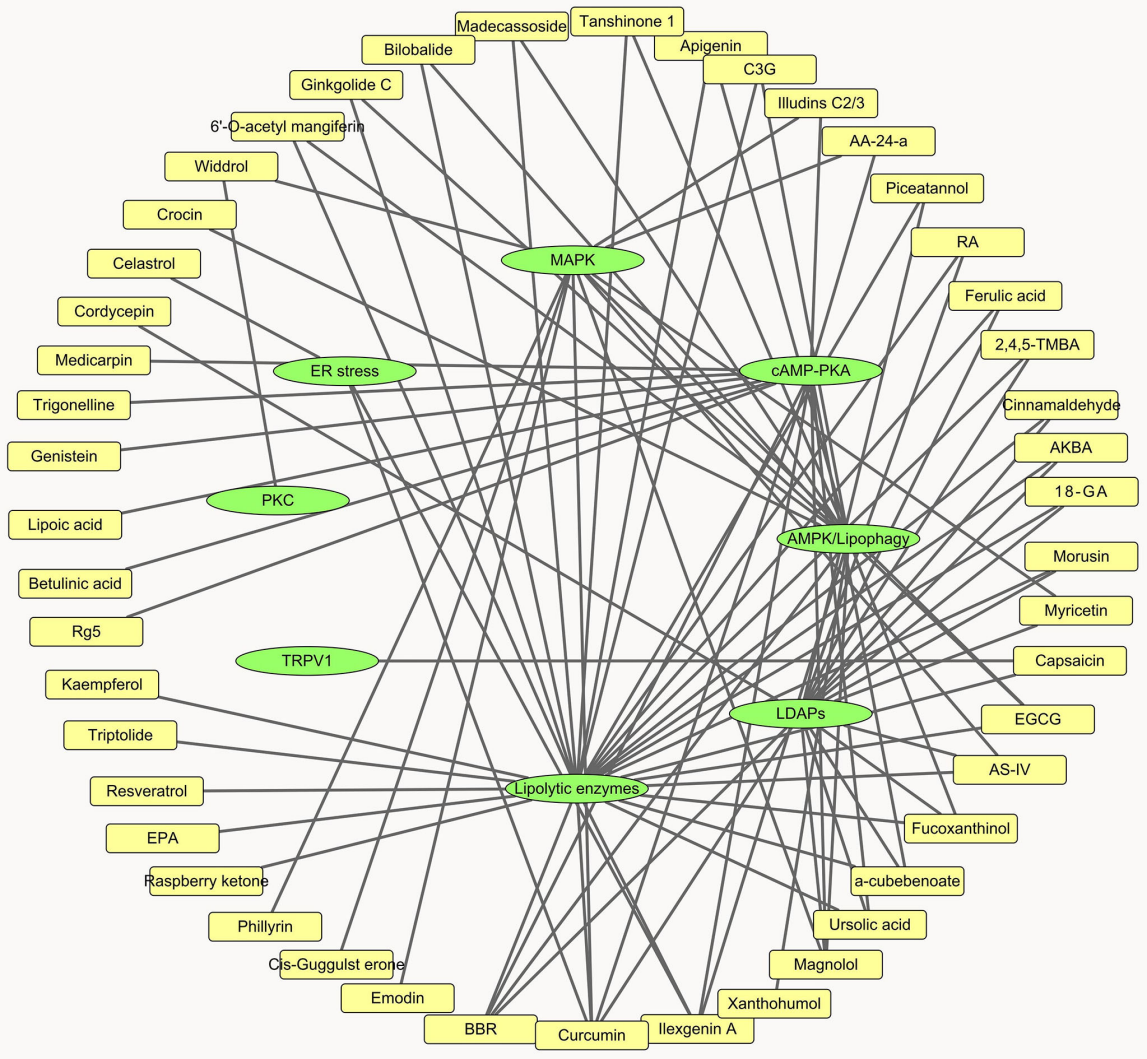
Network analysis of natural products and lipolytic pathways.

In the present review, we summarized the effect and modes of action of a wide range of natural products on lipolysis. Overall, these compounds individually or synergistically affect lipolytic enzymes, LDAPs, ER stress, and the cAMP-PKA, MAPK, AMPK, and PKC signaling pathways (**Figure 3**). The lipolytic effects of certain compounds have already been established. Nevertheless, their influences and mechanisms in fat synthesis and metabolism, their toxicity, and their effects on whole-body phenotypes, appetite, energy expenditure, and thermogenesis remain to be determined. About half the compounds evaluated herein affect lipolytic enzyme expression. However, *in vitro* enzyme activity assay and compound-enzyme interaction data were lacking for them. These experiments may help identify novel lipolysis inhibitors and agonists.

Our understanding of adipocyte lipolysis has progressed from basic knowledge of its associated enzymatic processes to elucidtation of the dynamic and complex regulatory mechanisms involved. Lipolysis interacts with other related processes, including thermogenesis, adipocyte browning, and lipogenesis. Clarification of the mechanisms of lipolysis and the changes it causes in whole-body energy metabolism has positive clinical value and socioeconomic benefits in that it may help develop modalities to prevent and treat obesity and its associated metabolic disorders. Lipolysis regulates TG metabolism and weight loss. Certain compounds with lipolytic activity, such as celastrol (141), apigenin (142), cordycepin (84), and BBR (143), have demonstrated anti-obesity efficacy. Theoretically, activating lipolysis may be a rational therapeutic approach for obesity. Thus far, however, no anti-obesity drugs targeting lipolytic enzymes or its related targets have been marketed.

The pathologies of obesity and its related metabolic conditions are highly complex. Simply targeting lipolysis can achieve weight loss. From the perspective of energy metabolism, however, weight loss is the result of multiple factors, including dietary restrictions and increases in lipolysis and energy utilization. It remains to be established whether lipolysis triggered by lipolytic agonists may damage certain cells, tissues, and organs or cause complications. The ideal anti-obesity drug should safely suppress appetite, increase lipolysis, and activate energy expenditure. Finally, the physiological functions of adipocytes should be rationally exploited, and their roles during metabolic disease should be identified. For patients with adipose dysfunction, the dynamic regulation of lipolysis and the amelioration of adipocyte dysfunction could improve obesity-associated metabolic conditions. For example, AS-IV and curcumin inhibit adipose lipolysis and thus prevent hepatic IR, which demonstrates their potential as treatments for metabolic-associated fatty liver disease through the regulation of lipolysis in adipose tissue during diseased states.

## Author contributions

X-DY and Y-YY wrote the manuscript. X-CG and S-YJ collected and checked the data. Y-YY contributions to design of the work and revised the work. All authors contributed to the article and approved the submitted version.

## Funding

This study was supported by the National Natural Science Foundation (No. 81603171) and the Natural Science Foundation of Hunan Province (2022JJ30860 and 2022JJ30862).

## Conflict of interest

The authors declare that the research was conducted in the absence of any commercial or financial relationships that could be construed as a potential conflict of interest.

## Publisher’s note

All claims expressed in this article are solely those of the authors and do not necessarily represent those of their affiliated organizations, or those of the publisher, the editors and the reviewers. Any product that may be evaluated in this article, or claim that may be made by its manufacturer, is not guaranteed or endorsed by the publisher.
